# Establishing RNAi in a Non-Model Organism: The Antarctic Nematode *Panagrolaimus* sp. DAW1

**DOI:** 10.1371/journal.pone.0166228

**Published:** 2016-11-10

**Authors:** Anna C. Seybold, David A. Wharton, Michael A. S. Thorne, Craig J. Marshall

**Affiliations:** 1 Department of Biochemistry, University of Otago, Dunedin, New Zealand; 2 Department of Zoology, University of Otago, Dunedin, New Zealand; 3 British Antarctic Survey, Natural Environment Research Council, Cambridge, United Kingdom; 4 Genetics Otago, University of Otago, Dunedin, New Zealand; James Hutton Institute, UNITED KINGDOM

## Abstract

The Antarctic nematode *Panagrolaimus* sp. DAW1 is one of the only organisms known to survive extensive intracellular freezing throughout its tissues. Although the physiological mechanisms of this extreme adaptation are partly understood, the molecular mechanisms remain largely unknown. RNAi is a method that allows the examination of gene function in a direct, targeted manner, by knocking out specific mRNAs and revealing the effects on the phenotype. In this study we have explored the viability of RNAi in *Panagrolaimus* sp. DAW1. In the first trial, nematodes were fed *E*. *coli* expressing *Panagrolaimus* sp. DAW1 dsRNA of the embryonic lethal genes *rps-2* and *dhc*, and the blister gene *duox*. *Pd-rps-2(RNAi)*-treated nematodes showed a significant decrease in larval hatching. However, qPCR showed no significant decrease in the amount of *rps-2* mRNA in *Pd-rps-2(RNAi)*-treated animals. Several soaking protocols for dsRNA uptake were investigated using the fluorescent dye FITC. Desiccation-enhanced soaking showed the strongest uptake of FITC and resulted in a significant and consistent decrease of mRNA levels of two of the four tested genes (*rps-2* and *tps-2a*), suggesting effective uptake of dsRNA-containing solution by the nematode. These findings suggest that RNAi by desiccation-enhanced soaking is viable in *Panagrolaimus* sp. DAW1 and provide the first functional genomic approach to investigate freezing tolerance in this non-model organism. RNAi, in conjunction with qPCR, can be used to screen for candidate genes involved in intracellular freezing tolerance in *Panagrolaimus* sp. DAW1.

## Introduction

The Antarctic nematode *Panagrolaimus* sp. DAW1 (formerly known as *Panagrolaimus davidi* CB1 and referred to here as PaDAW1 [1]) is the organism best documented to survive extensive intracellular freezing throughout its tissues [2]. The nematode is typically found associated with penguin colonies [3] and is exposed to episodic freezing and thawing during the summer, and long term freezing and cryoprotective dehydration during the winter [4–6]. The observation of recrystallization inhibition and hexagonal ice crystals in extracts of PaDAW1 [7] imply the presence of ice active proteins [8,9]. A number of attempts have been made to isolate ice-active proteins expected to play a key role in the survival of intracellular freezing [7], including a recent molecular analysis providing a comprehensive transcriptome and draft genome [10]. Whereas the isolation and characterisation of any ice-active proteins has so far proven elusive, the molecular work has enabled the beginning of functional genomic work in the nematode. Environmental RNA interference (RNAi) is a method developed in *C*. *elegans* [11] that allows the functional role of genes to be explored by causing the degradation of targeted mRNA, but does not work in every nematode species [12].

RNAi by feeding is a convenient reverse genetic technique that allows nematodes to develop under nearly natural conditions on a bacterial lawn [13]. The use of RNAi in nematode species other than *C*. *elegans* is often inefficient or inconsistent. In *Heligmosomoides polygyrus*, RNAi by feeding did not result in phenotypical changes or in significant down-regulation of overall mRNA levels [13]. Similar difficulties have been observed in *Haemonchus contortus* [14]. However, *Panagrolaimus superbus* and *Panagrolaimus* sp. PS1159, both showed embryonic lethal phenotypes following ingestion of *E*. *coli* expressing dsRNA for the *Panagrolaimus* embryonic lethal genes *ef1b* and *rps-2* [15]. Embryonic lethal phenotypes were also obtained in both species using dsRNA for *C*. *elegans* embryonic lethal genes *lmn-1* and *ran-4*.

Soaking nematodes in a dsRNA-containing solution is convenient and slightly more penetrative than feeding due to the addition of dsRNA uptake enhancing compounds. Although soaking seems to be the most efficient form of environmental RNAi, effects can still vary among genes, species and even experiments [14]. In *Bursaphelenchus xylophilus* a low but consistent knockdown (26%) was observed [16,17]. However, only five of eight genes in *Ostertagia ostertagi* [18] and two of 11 genes in *Haemonchus contortus* [14] were efficiently, but non-reproducibly, silenced. An extension of soaking has been used in mosquito larvae where osmotically-induced dehydration, followed by rehydration, has been shown to facilitate RNAi-uptake [19].

We investigated whether PaDAW1 is sensitive to high-throughput RNAi techniques such as feeding and soaking. RNAi by feeding was investigated in PaDAW1 using *E*. *coli* expressing dsRNA of the PaDAW1 homologues of the *C*. *elegans* embryonic lethal genes *rps-2* and *dhc*, and the blister gene *duox* [20]. RNAi by soaking in dsRNA solutions was investigated using several soaking protocols using both the fluorescent dye FITC and dsRNA of four different test genes. Finally, gene expression analysis on RNAi treated and non-treated nematodes was carried out using qPCR in order to evaluate the effect of gene silencing in each case. This first molecular approach could help to uncover the secret of intracellular freezing tolerance in PaDAW1, providing new insights in evolution and new applications for cryopreservation.

## Materials and Methods

### Nematode culturing and collection

PaDAW1 was originally isolated from McMurdo Sound region, Antarctica [3] in 1988 and has been maintained as a culture since then. Nematodes were grown on NGM agar plates at 20°C and sub-cultured weekly. Nematodes used for the experiments were collected within three days after sub-culturing using a modified Baermann technique [21], snap frozen in a mixture of dry ice and ethanol and stored at -80°C.

### RNA isolation and cDNA synthesis

RNA was extracted using TRIzol^®^ Reagent (Ambion, Foster City, CA, USA) and RNeasy^®^ Mini Kit (Qiagen, Hilden, Germany). Nematodes cooled on dry ice were first pulverized using a melted pipette tip as a pestle in a cold microcentrifuge tube, and then homogenized in TRIzol^®^ Reagent to ensure breakdown of the nematode cuticle. RNA was extracted and purified with the RNeasy^®^ Mini Kit according to the manufacturer's instructions. Purified RNA was quantified using a NanoDrop^®^ ND-1000 (Thermo Scientific, Boston, MA, USA) and a Qubit^®^ 2.0 Fluorometer (Life Technologies, Carlsbad, CA, USA) and reverse-transcribed using the VILO cDNA synthesis kit (Invitrogen, Carlsbad, CA, USA).

### RNAi probe and Primer design

Both the genomic DNA and cDNA sequences from PaDAW1 [10] were used to develop the RNAi probes and primers. To identify and target introns, as well as to avoid amplification of any contaminating genomic DNA, cDNA and genomic DNA sequences were aligned through in-house homology search tools and Spidey (http://www.ncbi.nlm.nih.gov/spidey/). Primers were designed by using Primer3web (http://primer3.ut.ee/), and potential primers were analyzed for possible dimer formation by using Beacon Designer Free Edition (http://www.premierbiosoft.com/) and for specificity by using NCBI Primer-BLAST (http://www.ncbi.nlm.nih.gov/tools/primer-blast/). Details of primers sequence and efficiency are shown in S1 File.

### Cloning

RNAi test genes (*rps-2*, *dhc*, *duox-42*) [22,23]were selected based on annotation through the PaDAW1 transcriptome [10] and homology with *C*. *elegans* genes reported to produce detectable phenotypes in RNAi experiments [15]. Shannon et al. reported successful RNAi for *rps-2* in two closely related *Panagrolaimus* species and so this gene was chosen for this work [15]. Another candidate gene was *tps-2a* (trehalose synthase [24]) as it is expected to play a significant role in cryoprotective dehydration and possibly intracellular freezing in PaDAW1 [10].

Target genes were PCR amplified from cDNA with gene specific primers using the Taq DNA Polymerase, dNTPack (Roche, Basel, Switzerland) (Table A in S1 File). PCR products were TA-cloned into the double IPTG inducible T7 RNA polymerase promoter vector pLitmus28i and their identity confirmed by sequencing. Plasmids with a perfect sequence match were either re-transformed into the *E*. *coli* HT115 feeding strain for RNAi-feeding or linearized using M13 PCR for in vitro transcription and RNAi-soaking.

### dsRNA production: Feeding

To prepare dsRNA for feeding experiments a modified protocol from Kamath and Ahringer was used [25]. The vector pLitmus28i containing the target gene was transformed into chemo-competent cells of the *E*. *coli* feeding strain HT115(DE3). Two PaDAW1 embryonic lethal genes (*Pd-rps-2* and *Pd*-*dhc*) and the blister gene (*Pd*-*duox-42*) were fed to both PaDAW1 and *C*. *elegans*. As a negative control dsRNA of the non-endogenous green fluorescent protein gene (*gfp*) was fed to the nematodes to assess for dsRNA toxicity.

A related experiment was also done where the *C*. *elegans* version of the three genes (*Ce-rps-2*, *Ce-dhc-1*, and *Ce-duox-2*) was fed to PaDAW1 and to *C*. *elegans*. A *gfp* negative control was also included in this analysis.

Feeding plates were prepared by adding 50 mg ampicillin, 0.5 mL tetracycline (25 mg/mL) and 240 μL 1 M IPTG to 1 L NGM agar medium to give final concentrations of ampicillin 50 μg/mL, tetracycline 12.5 μg/mL and IPTG 0.24 mM. Bacteria (grown for 6 h in liquid media with shaking at 37°C) containing each of the three test genes (*Pd*-*rps-2*, *Pd*-*dhc* and *Pd*-*duox*) were seeded into either separate 6 cm plates or 24 well plates (6 wells per gene) and allowed to grow for 12 h at ~20°C.

Nematodes were transferred to feeding plates using a stick with an attached eyelash. Approximately 100 L4 stage larvae were transferred from culture to an empty feeding plate where they were allowed to move thereby removing residual bacteria from the culture. The larvae were then transferred to the feeding plates containing bacteria expressing test genes and incubated at 22°C for 48 h.

The blistering phenotype associated with *duox* genes was assessed by counting 100 nematodes and noting any showing blisters. This count was done three times for each sample.

To screen for lethal phenotypes, three adult nematodes were transferred into each well of a 24 well plate containing bacteria expressed the appropriate gene (as above). Nematodes were allowed to lay eggs for 24 h and then removed. The eggs were incubated for 24 h (*C*. *elegans*) or 48 h (PaDAW1) and the total number of larvae hatched was assessed.

Each gene assessment was done three times in separate and independent experiments.

### dsRNA-production: Soaking

Double-stranded RNA for soaking experiments was produced by *in vitro* transcription of PCR products (M13 linearized pLitmus28i containing the target gene flanked by two T7 promoters) using the MEGAscript^®^ T7 Transcription Kit (Life Technologies, Carlsbad, CA, USA) according to the manufacturer's protocol. An annealing step was included, where ssRNA was incubated for 1 min at 90°C and then for 1 h at 37°C, to produce dsRNA, followed by a DNase step. The dsRNA was purified using phenol-chloroform extraction and ethanol precipitation. Purified dsRNA was dissolved in 50 μL soaking buffer (M9 buffer (22 mM KH_2_PO_4_, 42.3 mM Na_2_HPO_4_, 85.6 mM NaCl, 1 mM MgSO_4_), 0.05% gelatin and 3 mM spermidine, [26]), analyzed by NanoDrop spectrophotometry and agarose gel electrophoresis, and stored at -20°C.

### RNAi: Feeding

Approximately 100 L4 stage larvae were transferred to feeding plates as described for ‘dsRNA production: Feeding’ above.

After the initial feeding period, *duox*-fed nematodes were scored daily for blister phenotypes. To define the percentage of nematodes showing a blistering phenotype, three sets of 100 nematodes were scored.

To facilitate screening of embryonic lethal phenotypes, three adult nematodes, fed on bacteria containing embryonic lethal genes, were transferred into each of the six wells seeded with the same bacterial strain. Worms were allowed to lay eggs for 24 h and then removed. Eggs were allowed to hatch for 24 h or 48 h (*C*. *elegans* or PaDAW1) and the number of hatched larvae counted. Since PaDAW1 develops slower than *C*. *elegans*, PaDAW1 larvae were allowed to hatch 24 h longer. This same experiment was done three times to estimate the variability of these results.

### RNAi: Soaking

Three different soaking techniques were tested–soaking only, neurostimulant-, and desiccation-enhanced soaking–using the fluorescent dye fluorescein isothiocyanate (FITC) as a marker of uptake. For neurostimulant-enhanced soaking, 50 mM octopamine was added to the soaking solution. Approximately 30 mg nematodes were soaked in ~100 μL soaking solution containing 1 mg/mL FITC for 16 h at 20°C and then washed three times with ASTM type I water (mQ water). Fluorescence and viability of nematodes were analyzed immediately (0 h) and after 24 h, using an Olympus BX61 fluorescence microscope (Center Valley, PA, USA) and photographed using an Olympus DP71 camera.

**Fig 1:**
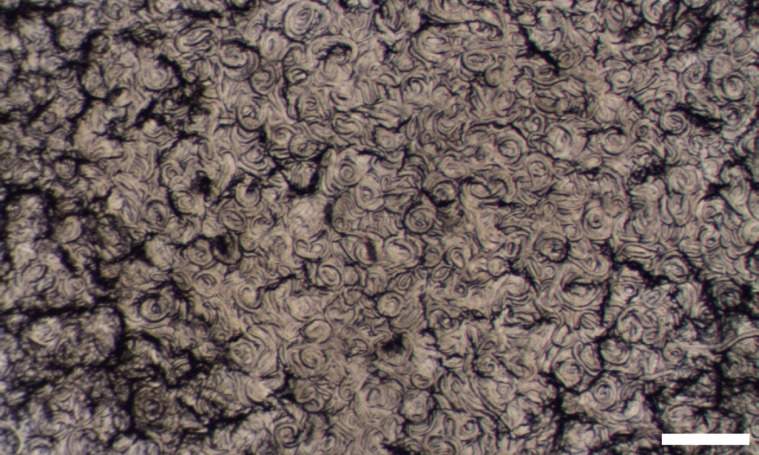
Micrograph showing a mass of coiled PaDAW1 desiccated for 24 h at 98% relative humidity. Scale bar, 1 mm.

Desiccation prior to soaking was tested to see if it enhanced dsRNA uptake. Nematodes were transferred to a microscope slide, surface water removed, and the slides were placed over a saturated potassium sulfate solution (reducing relative humidity to 98%) in a sealed container. Desiccation was monitored to assure that nematodes dried slowly enough to exhibit the typical coiled shape (Fig 1 characteristic of successful dehydration [27]. Nematodes were incubated for 24 h at 20°C and then rehydrated with soaking buffer containing 1 mg/mL dsRNA.

### Quantitative polymerase chain reaction (qPCR)

Quantitative PCR was performed using the BioRad CFX96 System (Hercules, CA, USA) and the BioRad SSoFast EVA Green Supermix with Low Rox. A 20 μL reaction contained 5 μL sample (total of 50 ng cDNA), 10 μL SYBR green mix, 1.2 μL primer mix and 3.2 μL mQ water. Primers were developed as described in ‘RNAi probe and Primer design’ above and details are shown in Table B in S1 File. Specificity and efficiency assays were performed for all genes [28]; details of these are shown in the supplementary information. Of six reference genes tested, the combination of *Pd-gpd-2* and *Pd-tba-1* (Table C in S1 File) were defined as the most stable and used for all qPCR experiments [28]. The BioRad CFX Manager was used to control qPCR settings and to analyze qPCR data.

### Data analysis

The qPCR data were analyzed using the BioRad CFX Manager. Means, standard deviations (s.d.), *P*-values and relative expressions of the normalized expression values were calculated in MS Excel. *P*-values were assessed using a t-test (parametric, two samples, equal variance). The ΔΔC_t_ (Livak) method was used to determine the relative difference in expression level of the target gene in different samples. In the first step, the C_t_ of the target gene was normalized to that of the reference gene, for both the test sample and the control sample (normalized relative expression value). Then, the ΔC_t_ of the test sample was normalized to that of the control sample (relative expression). This provides an estimate of how much expression of each of the test genes changed with respect to the control condition. This approach corrects for differences in the amount of RNA extracted from each culture which would otherwise skew these data.

Statistical analyses were done using R [29] and the program RStudio. Details of each statistical analysis for each figure are shown in the supplementary information (S2–S5 Files).

## Results

### Investigating RNA-Feeding: Phenotypic analysis

To test whether PaDAW1 is accessible to the RNAi feeding technique, two embryonic lethal genes in *C*. *elegans* were targeted. We used the PaDAW1 and *C*. *elegans* homologues of *rps-2* and *dhc* (*Pd-rps-2* and *Pd-dhc*, and *Ce-rps-2* and *Ce-dhc-1* respectively) fed to both PaDAW1 and *C*. *elegans* to test whether a cross-species RNAi effect could be detected similar to that reported in two other *Panagrolaimus* species and *C*. *elegans* [15].

**Fig 2:**
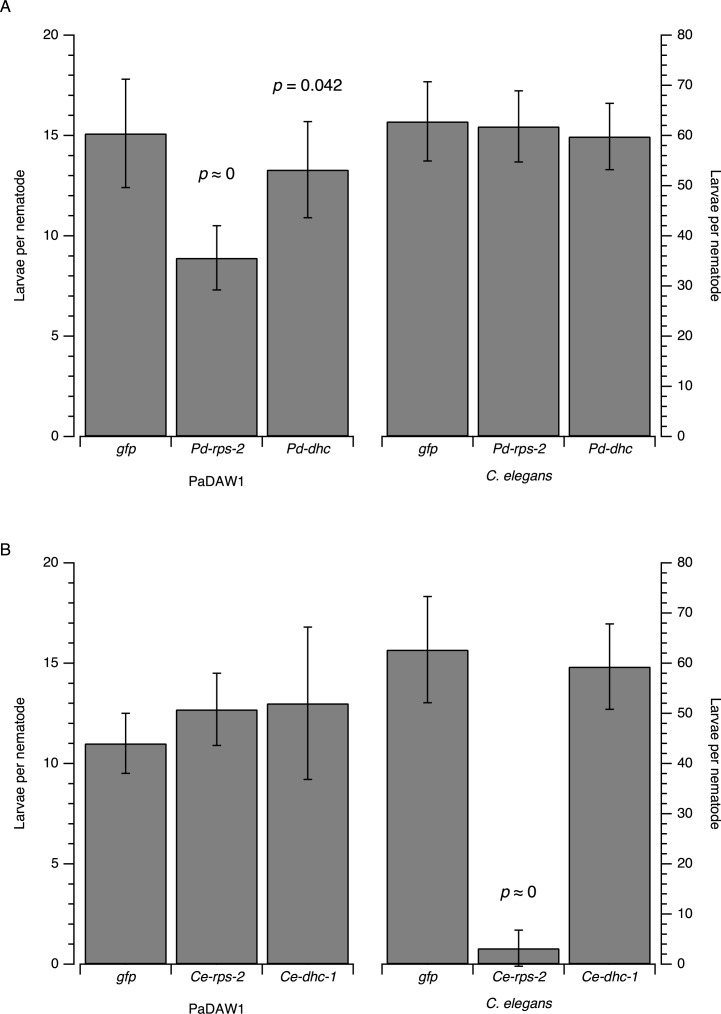
Embryonic lethal phenotypes in *C*. *elegans* and PaDAW1 after feeding *E*. *coli* expressing dsRNA. (A) Larval hatching of *gfp(RNAi)*-treated, *Pd-rps-2(RNAi)*-treated and *Pd-dhc(RNAi)*-treated PaDAW1 (left y-axes) and *C*. *elegans* (right y-axes) 48 h after removal of adults from the plates. Each value represents the mean±s.d. of 6–18 biological replicates (three nematodes per biological replicate). For PaDAW1 the experiment was replicated three times (six biological replicates per experiment) and the values represent the mean±s.d. of all 18 biological replicates. (B) Larval hatching of *gfp(RNAi)*-treated, *Ce-rps-2(RNAi)*-treated and *Ce-dhc-1(RNAi)*-treated PaDAW1 (left y-axes) and *C*. *elegans* (right y-axes) 24 h after removal of adults from the plates. Each value represents the mean±s.d. of 6 biological replicates. Conditions where a significant decreases in larval hatching was assessed by multiple comparison of means by Dunnett Contrasts [30] are indicated with a *p* value (see S2 File).

Phenotypic analysis of embryonic lethal genes showed a significant reduction in larval hatching in *Pd-rps-2(RNAi)* treated PaDAW1 compared to the *gfp(RNAi)* treated controls (Fig 2A). *Pd-dhc(RNAi)* treated PaDAW1 showed a smaller decrease in larval hatching compared to the controls (*p* = 0.042) that is probably not biologically significant. No increase in embryonic lethality was observed in *C*. *elegans* fed on PaDAW1 genes (Fig 2A).

For PaDAW1, three independent experiments were performed to ensure reproducibility of results. In all experiments, *Pd-rps-2(RNAi)* treated PaDAW1 showed a significant decrease in the number of hatched larvae. A non-significant decrease in eggs laid was also observed with *Pd-rps-2(RNAi)* treated animals suggesting that hatching was affected rather than egg production.

A *C*. *elegans* control was used to assess this technique. The *C*. *elegans* embryonic lethal genes *Ce-rps-2* and *Ce-dhc-1* were fed to *C*. *elegans* as well as to PaDAW1 (Fig 2B). Phenotypic analysis showed a significant reduction in hatched larvae in *Ce-rps-2(RNAi)* treated *C*. *elegans* compared to the *gfp(RNAi)* treated controls (*P* < 0.001) (Fig 2B). However, *Ce-dhc-1(RNAi)* treated *C*. *elegans* showed no significant decrease in hatched larvae (*P* = 0.827). The cross-species experiment showed no significant effect on larval hatching in PaDAW1 fed either *C*. *elegans* or PaDAW1 dsRNA.

There was an obvious difference in the number of eggs laid between the two nematode species. Controls showed that in a period of 24 h, *C*. *elegans* lay about 4× more eggs (~60 eggs) than PaDAW1 (~15 eggs).

To test high throughput techniques in PaDAW1, very clear phenotypes are required. In *C*. *elegans*, mutations in the *duox-2* gene led to an obvious blister phenotype [31]. An RNAi experiment was performed to test the effect of *Pd-duox-42* in PaDAW1 and (as a positive control) the homologous *Ce-duox-2* in *C*. *elegans*.

Cross-species RNAi was done in parallel by feeding *Ce-duox-2* to PaDAW1 and *Pd-duox-42* to *C*. *elegans*. The results show a high percentage (70%) of *C*. *elegans* nematodes with a blistering phenotype when fed *Ce-duox-2* (S1 Fig). However, no such phenotype was observed in PaDAW1 or in the cross-species RNAi experiments in either *C*. *elegans* or PaDAW1 (S1 Fig).

### Investigating RNAi-Feeding: Gene expression analysis

**Fig 3:**
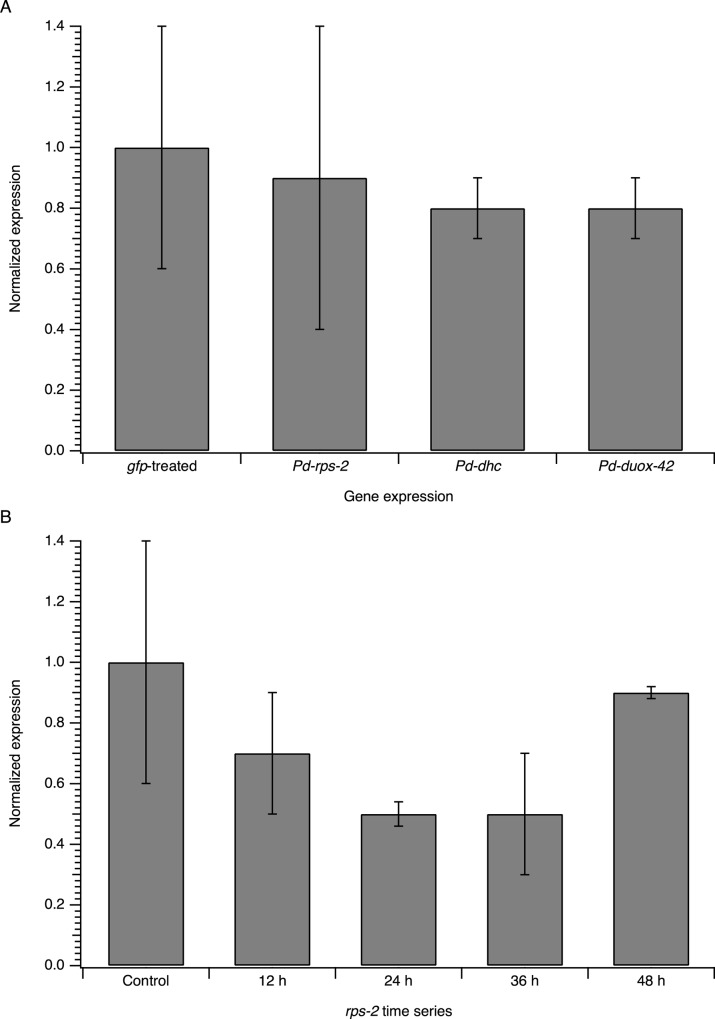
Gene expression analysis of RNAi-feeding in PaDAW1. (A) qPCR of *gfp(RNAi)*-treated (control) *Pd-rps-2(RNAi)*, *Pd-dhc(RNAi)* and *Pd-duox-42(RNAi)* treated PaDAW1 (B) qPCR of *Pd-rps-2(RNAi)* after a feeding period of 12 h, 24 h, 36 h and 48 h. The expression of RNAi treated samples is shown relative to control samples, which are normalized to a value of 1. Each value represents the mean±s.d. of three or four biological replicates. No significant differences were found in these data (see S3 File).

To investigate the results obtained by phenotypic analysis and to assess whether gene expression was reduced despite the absence of a phenotype in PaDAW1, expression analyses were done by qPCR. RNA was isolated from *gfp*-treated (control), *Pd-rps-2*, *Pd-dhc* and *Pd-duox-42* treated PaDAW1 after a feeding period of 48 h at 22°C. For *gfp*-treated, *Pd-rps-2* and *Pd-dhc* treated samples, four replicates and for *Pd-duox-42* three replicates were collected. Data were normalized to the control (relative expression = 1.0), showing the change relative to the control. The results show that there is no significant down-regulation for any of these genes after 48 h (Fig 3A). Notable is the large biological variation within replicates, especially of the non-treated and the *Pd-rps-2(RNAi)* treated samples.

The lack of down-regulation of *Pd-rps-2* compared to the non-treated control was surprising after a small but significant decrease in larval hatching was seen in the phenotypic analysis. The lack of effect might reflect that the 48 h sampling time lay outside the period of *rps-2* down-regulation. To test this possibility, a time series of the *Pd-rps-2* (RNAi) was done by sampling nematodes after feeding periods of 12 h, 24 h, 36 h and 48 h (three replicates for each time). The expression of *Pd-rps-2* after different feeding periods relative to the control is shown in Fig 3B. The time series shows a trend with the greatest down-regulation after 24 h of feeding. However, there was no statistically significant difference in mRNA level between the 24 h sample and the control assessed using a repeated measures ANOVA.

**Fig 4:**
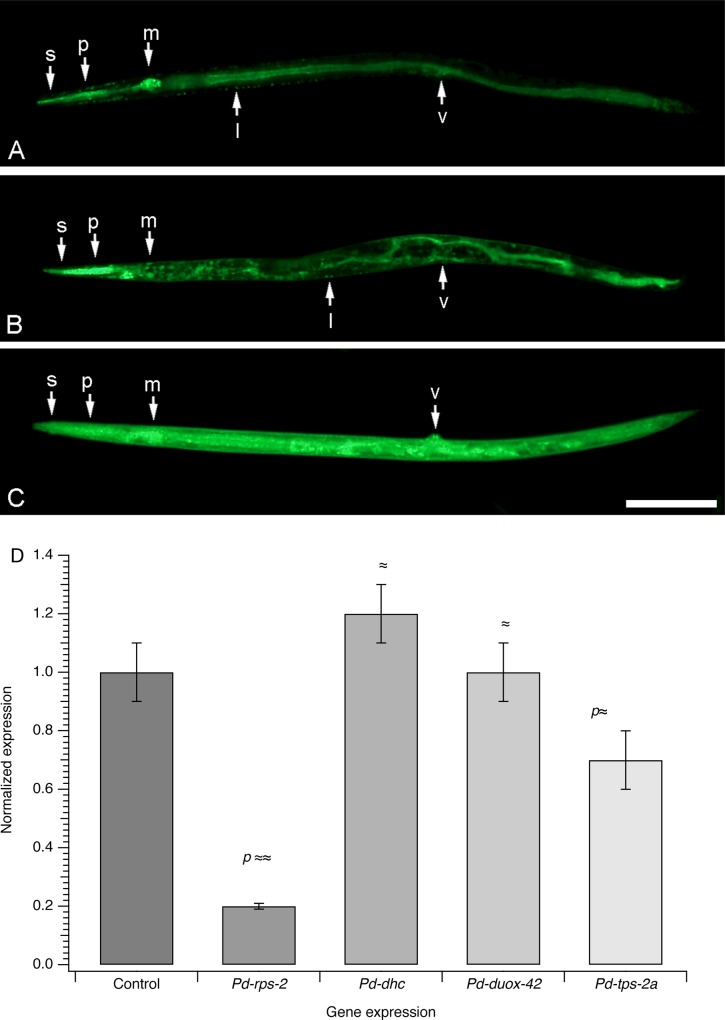
Phenotypic analysis of three different RNAi-soaking techniques. (A-C) Micrographs showing the FITC uptake in PaDAW1 using three different soaking techniques: soaking-only (A), octopamine-enhanced soaking (B) and desiccation-enhanced soaking (C). s = stylet, p = pharynx, m = median bulb, l = lipids and v = vulva. Scale bar, 0.1 mm. (D) Survival rate of nematodes for soaking-only, octopamine-enhanced soaking and desiccation-enhanced soaking. Each value represents the mean±s.d. of 4 biological replicates and an analysis of variance showed no significant differences among conditions (see S4 File).

We did note that the variability of the mRNA expression in these experiments was large (Fig 4), despite careful standardization of the experimental protocol and three biologically separate analyses. This variability might mask a small effect, but these data suggest an absence of any large effect of RNAi on mRNA expression in this experimental system.

### Investigating RNAi-Soaking: Phenotypic analysis

To develop a method to perform RNAi soaking in PaDAW1, we needed to show that the soaking solution, and thus dsRNA, was taken up by live and viable nematodes. The fluorescent dye FITC was used to monitor soaking solution uptake in three different soaking approaches (soaking-only, along with octopamine-, and desiccation-enhanced soaking).

All three techniques resulted in a clear accumulation of fluorescence in the nematodes not seen in the controls soaked without FITC. Nematodes soaked without FITC showed a weak and diffuse gut fluorescence. Whereas soaking-only and octopamine-enhanced soaking resulted in localized FITC accumulation including the epidermal region (Fig 4A and 4B), desiccation-enhanced soaking resulted in a stronger uptake into the surrounding tissue (Fig 4C) where nematodes showed green fluorescence mainly in the gut with strong signals in the median bulb and the vulva.

To define conditions for optimal uptake of dsRNA (maximal uptake with minimal lethality), several soaking conditions were tested by using the fluorescent dye FITC. The number of active and inactive nematodes was determined by counting three sets of 100 animals in each of four replicates. Fig 4D shows the survival of nematodes treated with soaking-only, octopamine- and desiccation-enhanced soaking 24 h after the soaking solution was washed off. Nematodes treated by soaking-only showed 23% inactivity, those treated with octopamine-enhanced soaking showed 17% inactivity and nematodes desiccated prior to soaking showed 32% inactivity. In general, more strongly fluorescent nematodes were less active suggesting that the soaking solutions were deleterious to the nematodes.

### Investigating RNAi-Soaking: Gene expression analysis

**Fig 5:**
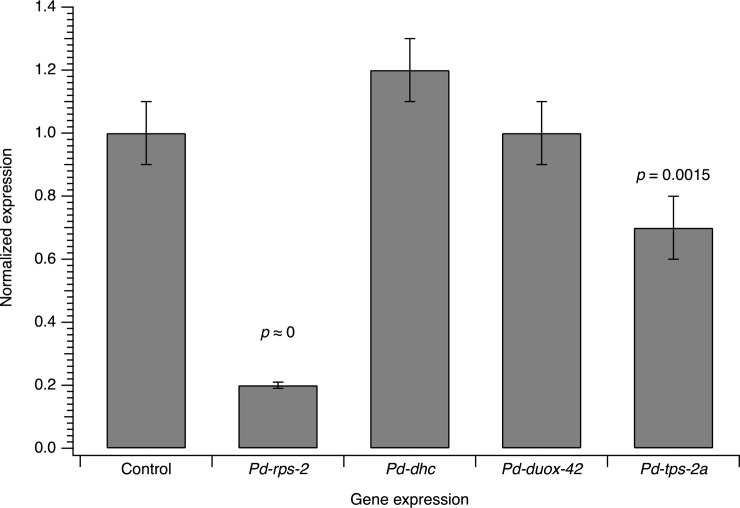
Gene expression analysis of RNAi-soaking in PaDAW1. qPCR of *Pd-rps-2(RNAi)*, *Pd-dhc(RNAi)*, *Pd-duox-42(RNAi)* and *Pd-tps-2a(RNAi)* treated samples relative to the control (non-treated sample). The control is shown as normalized to a value of 1 and the values for each gene indicate the expression relative to the control. Each value represents the mean±s.d. of three or four biological replicates. For *Pd-rps-2(RNAi)* and *Pd-tps-2a(RNAi)* the experiment was repeated (three or four biological replicates per experiment) and the values represent the mean±s.d. of all six or eight biological replicates. Significant differential expression (assessed using a Dunnett contrasts test) is indicated by the relevant *p* value (see S5 File).

To test whether desiccation-enhanced soaking led to systemic uptake of dsRNA and subsequently to silencing of the target gene, qPCR was performed on nematode extracts after they were soaked for 16 h followed by a 24 h incubation to allow the processes associated with RNAi to occur. Fig 5 shows the mRNA amount of four different genes, *Pd-rps-2*, *Pd-dhc*, *Pd-duox-42* and *Pd-tps-2a* relative to the non-treated control. Compared to the controls, the mRNA level of *Pd-rps-2(RNAi)* treated nematodes was strongly and significantly reduced (relative expression 0.21, *p* ≈ 0.000) and that of *Pd-tps-2a(RNAi)* treated nematodes was slightly and significantly reduced (relative expression 0.72, *p* = 0.0015). However, *Pd-dhc(RNAi)* and *Pd-duox-42(RNAi)* treated samples did not show any significant change in mRNA expression compared to the control. For *Pd-rps-2* and *Pd-tps-2a*, two independent experiments were performed to ensure the validity of results.

## Discussion

### RNAi in PaDAW1

The use of RNAi in non-model organisms has proven effective as a way of investigating interesting physiological and biochemical states. Previous work suggested that environmental feeding technique developed for *C*. *elegans* is effective in another *Panagrolaimus* species, *P*. *superbus*, despite the substantial phylogenetic gap [15] and the difficulties of transferring this approach to other species of *Caenorhabditis* [32]. Our initial work sought to replicate the work of Shannon et al. [15]. We were not successful using that approach and so we developed another strategy to allow us to use RNAi in PaDAW1.

### Investigating RNAi-Feeding: Phenotypic analysis

The protein RPS-2 plays a key role in protein translation and mutations lead to embryonic lethality in *C*. *elegans*. For this gene, we observed a clear phenotype in both PaDAW1 and *C*. *elegans* (Fig 2). We were able to show a reduction in mRNA production after desiccation-enhanced soaking when assessed by qPCR in PaDAW1 (Fig 5). In *C*. *elegans*, *Ce-rps-2* is known to cause both a sterile maternal RNAi phenotype [25] and an embryonic lethal RNAi phenotype [33]. RNAi using *rps-2* from *Panagrolaimus sp*. *PS1159* was shown to cause 80% embryonic lethality in *P*. *superbus* and 38% embryonic lethality in *Panagrolaimus sp*. *PS1159*, but no maternal sterility [15]. Our results show that the production of larvae was repeatedly decreased by ~50% for RNAi using *Pd-rps-2* in PaDAW1 –and not as great as the effect in *C*. *elegans* (Fig 2).

In order to trigger cross-species RNAi successfully, a minimum sequence match of approximately 19 bp (the length of a siRNA) is considered canonical [34]. A sequence comparison between PaDAW1 and *C*. *elegans* genes revealed that this requirement is not met and this probably explains the lack of phenotype. This requirement was met for genes from *H*. *polygyrus* and *C*. *elegans*, explaining the successful cross-species RNAi experiment reported by Lendner et al. [13]. That cross-species RNAi was successful between *P*. *superbus* (a close relative to PaDAW1) and *C*. *elegans* [15] is surprising in the light of our findings and the general view that a 19 bp identity is required for RNAi to be successful [34]. It was not possible to assess the match between RNAi constructs and the *P*. *superbus* genes from the data provided in Shannon *et al*. [15].

To test high-throughput techniques such as feeding in PaDAW1, very obvious phenotypes were required. In *C*. *elegans*, DUOX-2 catalyses the cross-linking of tyrosine residues involved in the stabilization of cuticular extracellular matrix and mutations here lead to an obvious blistering phenotype [31]. Our results show a high percentage (70%) of *C*. *elegans* nematodes with the typical blistering phenotype. However, no such phenotype could be observed with PaDAW1. This could be due to the fact that *Pd-duox-42* is not efficiently silenced by RNAi or that DUOX-42 has a different function in PaDAW1 and hence mutations do not lead to a blistering phenotype.

### Investigating RNAi-Feeding: Gene expression analysis

Gene expression analysis by qPCR was performed in order to confirm the results obtained by phenotypic analysis. Our preliminary results showed no significant down-regulation for any of the tested genes after a feeding period of 48 h. For *Pd-rps-2* this result was surprising, since a clear phenotype—a reduction in larval hatching—suggested that RNAi on *Pd-rps-2* had worked. Previous experiments showed a lack of phenotype despite a down-regulation of the target gene suggesting that RNAi may have an effect even though no phenotype is evident [35]. However, it is unlikely that there is a phenotype without a measurable decrease in the corresponding mRNA.

The significant biological variation we observed within replicates confounds attempts to detect variation between treatments. This biological variation could result from small differences in the handling of nematode cultures or molecular techniques, or be intrinsic to nematode growth. We did find that this variation was reduced by transferring an exactly equal volume of nematode culture to each replicate plate and storing them next to each other (instead of above each other). Small differences in RNA extraction (e.g. homogenization technique) might also lead to technical variation within the biological replicates. To improve this, replicate RNA was extracted from exactly the same amount of nematode tissue and cDNA was synthesized from exactly the same quality and quantity of RNA. However, the use of these approaches did not reduce experimental variability any further.

The lack of down-regulation could also be due to the sampling time of 48 h falling outside the time of effective down-regulation. The *Pd-rps-2* time-series indicates that, in common with *C*. *elegans*, 24 h is sufficient to observe maximum gene silencing in PaDAW1 (Fig 3B). However, although feeding is the least invasive technique, it appears to be incapable of producing a robust and consistent phenotype in PaDAW1. Work in other nematode species has already shown that environmental RNAi may be of variable success [12,13]. Geldhof et al. [14] tested three different delivery techniques in *H*. *contortus*: feeding, soaking and electroporation. While no phenotype or decreased mRNA level was obtained with feeding, soaking efficiently silenced two of 11 genes tested and electroporation two of four genes.

These results show that although the applied feeding technique is capable of inducing a phenotype in *C*. *elegans*, it is not consistent and does not fulfill the requirements of a reliable screening technique in PaDAW1.

### Investigating RNAi-Soaking: Phenotypic analysis

The dsRNA uptake of three different soaking techniques was monitored by using the fluorescent dye FITC. We investigated three approaches: soaking-only, octopamine-, and desiccation-enhanced soaking. All three techniques resulted in distinct FITC accumulation in the nematodes that was not observed in controls where weak and diffuse gut fluorescence is probably autofluorescence [14]. Nematodes soaked both with and without octopamine showed mainly localized fluorescence, whereas nematodes desiccated prior to soaking consistently showed fluorescence throughout their bodies.

Nematodes soaked with and without octopamine showed localized fluorescence along the gut, with strong signals in the median bulb and vulva, suggesting uptake from the body openings [26]. Significant FITC accumulation in the pharynx and median bulb indicates an uptake via the mouth and fluorescence in the epidermal region suggests that FITC enters via the cuticle. Localized fluorescence indicates that FITC is taken up only into tissues directly exposed to the environment without a systemic spread into more distal regions. The small difference between nematodes soaked with and without octopamine suggests that PaDAW1 accumulates FITC locally without neurochemical stimulation [26].

In contrast to the localized uptake, desiccation-enhanced soaking resulted in a stronger signal in the surrounding tissue, indicating widespread cellular uptake. It is likely that after a desiccation period of 24 h, nematodes are dehydrated both extra- and intra-cellularly, and therefore the rehydrating soaking solution is taken up by all cells in PaDAW1. Osmotically-induced dehydration, followed by rehydration has been shown to facilitate RNAi by soaking in mosquito larvae [19].

Optimal dsRNA uptake is not only defined by maximal uptake of the soaking solution (and dsRNA) but also by minimal lethality. Nematodes desiccated prior to soaking showed the strongest fluorescence but also the highest inactivity (32%). However, a positive correlation between desiccation rate and survival was observed, with slowly-desiccated nematodes showing a coiling behaviour that correlated with higher activity. This observation is consistent with the observation by Wharton and Barclay [27] that indicates PaDAW1 is an external dehydration strategist, requiring a slow rate of water loss to survive desiccation [36]. Controlled slow desiccation was used to further improve survival of this desiccation-enhanced RNAi soaking technique.

### Investigating RNAi-Soaking: Gene expression analysis

The down-regulation of *rps-2* and *tps-2a* in PaDAW1 indicates that the dsRNA preparations and culture conditions used are capable of silencing a target gene in this nematode. Desiccation prior to soaking enhances the uptake of dsRNA-containing soaking solution at a cellular level. Our finding that desiccation-enhanced soaking reduced the *Pd-rps-2* level significantly and repeatedly shows its stronger penetrance compared to the feeding technique. However, the fact that only two genes of the four tested were down-regulated indicates that although the RNAi pathway is functional and RNAi under certain conditions is possible, not all genes are susceptible. Limited success of environmental RNAi has been shown in other nematode species. In *H*. *contortus* two of 11 tested genes were successfully silenced and in *O*. *ostertagi* it was five of eight [14,18]. Visser et al. [18] demonstrated that some genes are more sensitive to RNAi than others due to factors such as different locations, stability, transcript level and/or secondary structure of the mRNA.

In *C*. *elegans*, the two transmembrane proteins SID-1 and SID-2 are required for dsRNA uptake and spread. Species such as *C*. *briggsae*, lacking functional SID-1 and SID-2, are recalcitrant to environmental RNAi [32]. A recent screening of the PaDAW1 genome did not confirm the presence of these proteins, possibly explaining the limited efficiency of RNAi [10]. However, Kushwaha et al. [37] reported that *B*. *malayi* genes can be efficiently silenced by RNAi despite the lack of SID-1 and SID-2 in this species, suggesting the involvement of other genes. This alternative entry and spread, however, might not be as efficient and may also explain the varying RNAi efficiency we observed in PaDAW1. In addition, RNAi also depends on technical variations such as dsRNA concentration, base composition, dsRNA position on the target gene as well as dsRNA sequence length [26].

Our data show that desiccation-enhanced soaking significantly and repeatedly down-regulated two of four genes, indicating that the dsRNA preparations and the culture conditions used are efficient. Desiccation prior to soaking enhances the take-up of the dsRNA-containing solution and results in a stronger phenotype when compared to feeding. However, the fact that only two genes were affected stresses the importance of confirming the down-regulation of each target gene by qPCR in order to know whether or not RNAi has been successful. This further emphasises the importance of clear phenotypes as RNA knockdown does not of itself necessarily lead to the desired physiological effect. The desiccation-enhanced soaking technique presented here can be used–in conjunction with qPCR–to screen for candidate genes associated with this nematode's remarkable freezing survival abilities. Establishing a tool such as RNAi in a non-model system is possible but careful consideration should be made of the work involved in transferring even simple procedures from model organisms. Recent work using the CRISPR/Cas9 system in the nematode *Pristionchus pacificus* [38] suggests this might be a fruitful approach for examining gene function in PaDAW1. Carefully-targeted gene knockouts could assess the function of specific genes (and combinations of genes). This is likely to be particularly useful in a parthenogenic species like PaDAW1 [39] where the absence of sexual reproduction (and thus recombination of lines) hinders conventional genetic analysis.

## Supporting Information

S1 Fig Blister phenotypes in *C*. *elegans* and PaDAW1. This figure shows the percentage of blistering phenotypes in *Ce-duox-2(RNAi)* treated *C*. *elegans* and in *Pd-duox-42(RNAi)* treated PaDAW1, as well as in cross-species RNAi. Each value represents the mean±s.d. of three biological replicates.
(PDF)

S1 File qPCR Supplementary Information. Data relating to PCR and qPCR including primers sequences, efficiency plots, and supplementary tables A, B and C, and supplementary figures A and B.
(DOCX)

S2 File Statistical data for Fig 2. (TXT)

S3 File Statistical data for Fig 3. (TXT)

S4 File Statistical data for Fig 4. (TXT)

S5 File Statistical data for Fig 5. (TXT)
