# Supplementary figures and images for: Establishing RNAi in a Non-Model Organism: The Antarctic Nematode Panagrolaimus sp. DAW1
Source: PLoS One. 2016 Nov 10;11(11):e0166228. doi: 10.1371/journal.pone.0166228 (PMC5104476; doi:10.1371/journal.pone.0166228)

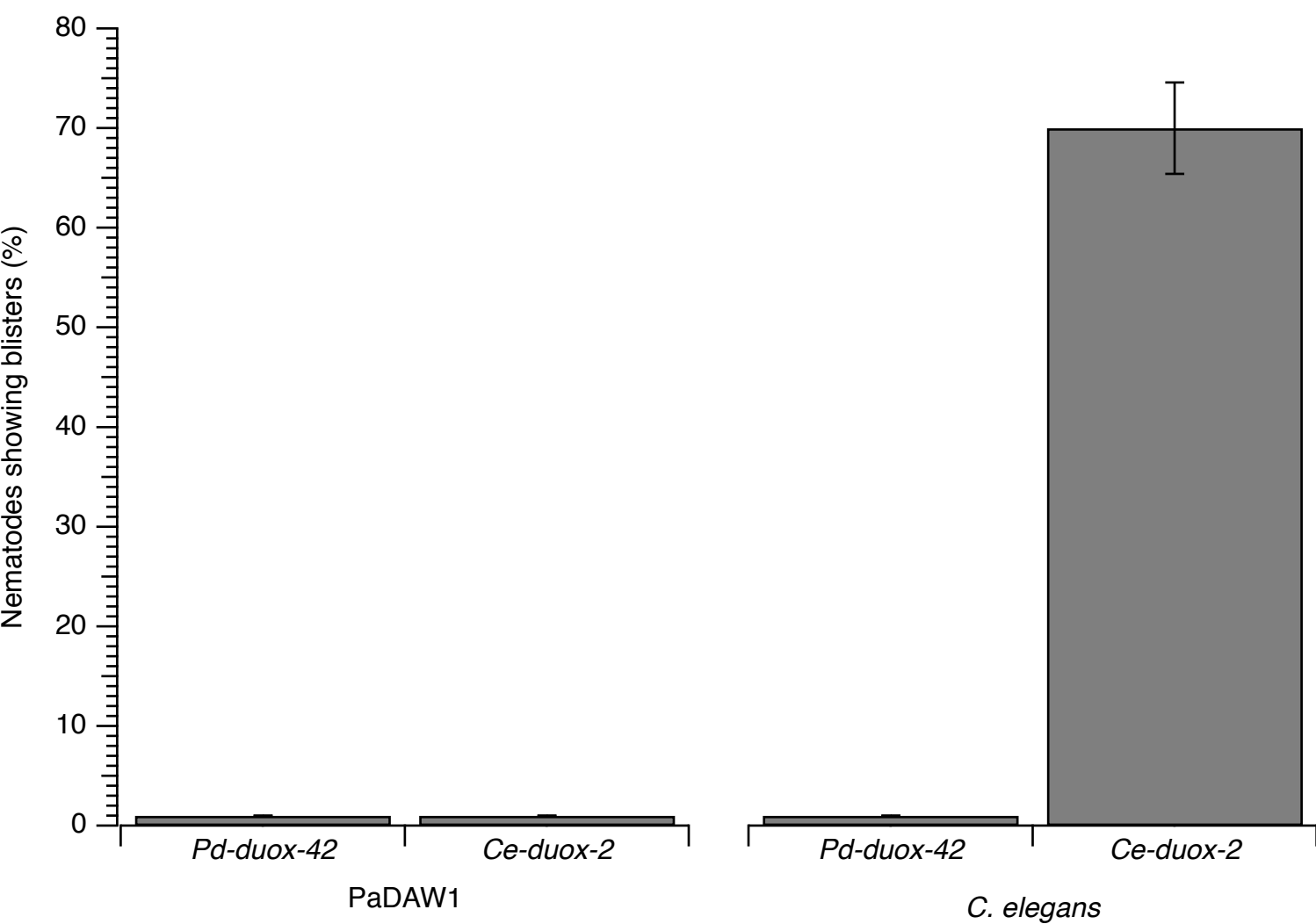

Supplement: S1 Fig — This figure shows the percentage of blistering phenotypes in Ce-duox-2(RNAi) treated C. elegans and in Pd-duox-42(RNAi) treated PaDAW1, as well as in cross-species RNAi. Each value represents the mean±s.d. of three biological replicates. (PDF) [file pone.0166228.s001.pdf]
