# Supplementary material for: Establishing RNAi in a Non-Model Organism: The Antarctic Nematode Panagrolaimus sp. DAW1
Source: PLoS One. 2016 Nov 10;11(11):e0166228. doi: 10.1371/journal.pone.0166228 (PMC5104476; doi:10.1371/journal.pone.0166228)
Supplement: S1 File — Data relating to PCR and qPCR including primers sequences, efficiency plots, and supplementary tables A, B and C, and supplementary figures A and B. (DOCX) [file pone.0166228.s002.docx]

**PCR Primers for amplification of genes for dsRNA production**

| Primer name | Sequence (5' to 3') | Tm (°C) | Start (bp) | End (bp) | Clone  size (bp) | cDNA size (bp) | Target  intron (bp) | Pd number |
| --- | --- | --- | --- | --- | --- | --- | --- | --- |
| *Pd-dhc* F | GTGCTTTGTGGCCCACCGGG | 75.9 | 1 | 440 | 440 | 4020 | 294 | PdU054220_v1.1 |
| *Pd-dhc* R | TAATCAACATAAACAACTGG | 52.5 |  |  |  |  |  |  |
| *Pd-duox-42* F | TTTTGGCAAGAAGGTGATCC | 63.9 | 1746 | 2130 | 385 | 4925 | 2088 | PdU000050_v1.1 |
| *Pd-duox-42* R | CGGTAACAGAACGGCAAAAT | 63.6 |  |  |  |  |  |  |
| *Pd-rps-2* F | TGGTCATGTTGGTCTTGG | 60.6 | 486 | 928 | 443 | 1018 | 633 | PdU054967_v1.1 |
| *Pd-rps-2* R | GACGAACAGGTGCTGTTGAA | 64.0 |  |  |  |  |  |  |
| *Pd-tps-2a* F | TGCTTGAGTTTGTGAATCTGGG | 66.2 | 788 | 1321 | 534 | 1564 | 1213 | PdU054198_v1.1 |
| *Pd tps-2a* R | ACGCTACAAGTTTATCATCCAG | 59.7 |  |  |  |  |  |  |

**Table A:** Genes and PCR primers used to amplify PaDAW1 genes for dsRNA production in *E. coli*. In addition to the gene names and primers sequences, primer melting points, clone location and size, cDNA size, target intron location, and the PaDAW1 contig name [1] is shown. The cDNA size refers to the size of the gene identified at each Pd number.

#### qPCR primers

qPCR primers were designed and prepared as described for PCR primers, except the product size range was set to 80-120 bp. Although the maximum temperature difference of primer pairs was set to 1 °C using Primer3web, the final temperature difference of some primer pairs exceeded this value. Sequences for all qPCR primers are shown in Table S2.

| Primer name | Sequence (5' to 3') | Tm (°C) | Use | Efficiency (%) |
| --- | --- | --- | --- | --- |
| *Pd-dhc* F | TCAATACACTTTTGGCAAATGGA | 65.0 | qPCR validation | 89.2 |
| *Pd-dhc* R | TGAGTCAAGCATTAAACCAGACT | 61.6 | qPCR validation | 89.2 |
| *Pd-duox-42* F | TATTCATCCTCGACGACCTT | 61.1 | qPCR validation | 88.8 |
| *Pd-duox-42* R | TTAGCGACATCCAATTTGCT | 62.1 | qPCR validation | 88.8 |
| *Pd-gpd-2* F | TGTGTTGCTGTTAATGATCCTT | 61.0 | Reference gene | 98.4 |
| *Pd-gpd-2* R | CAAGATTTCCACCTTCTGCTTT | 63.2 | Reference gene | 98.4 |
| *Pd-rps-2* F | TCGTCTTGTTAAAGAACGCAAA | 63.4 | qPCR validation | 104.4 |
| *Pd-rps-2* R | AACTTCATCACGGAGATTAGGG | 63.0 | qPCR validation | 104.4 |
| *Pd-tba-1* F | CCGAGGGGATGTTGTACCTA | 63.6 | Reference gene | 95.6 |
| *Pd-tba-1* R | TAAAGCCAGTTGGACACCAAT | 63.1 | Reference gene | 95.6 |
| *Pd-tps-2a* F | AGTTTCAGAATCACCACAGACG | 63.1 | qPCR validation | 109.3 |
| *Pd-tps-2a* R | TGATAAAGGGCAGGGCATTG | 66.9 | qPCR validation | 109.3 |

**Table B:** Names, sequences, use and efficiency of qPCR primers. Primer names are formed according to the following rules: Species name (e.g. *Pd* = PaDAW1*,* - gene (e.g. *dhc*) - primer (F = forward, R = reverse).

#### Specificity and efficiency assays

Specificity assay: To ensure that primers amplify the expected product size range of 80-120 bp and that there was no amplification from any contaminating genomic DNA, primers were tested using standard PCR. PCR products were analysed via agarose gel electrophoresis for primer specificity (single band of the expected size), for genomic DNA contamination (no band in the -RT control and no or a bigger band in the gDNA control) and general contamination (water only control). Figure S1 presents an example of such an agarose gel.


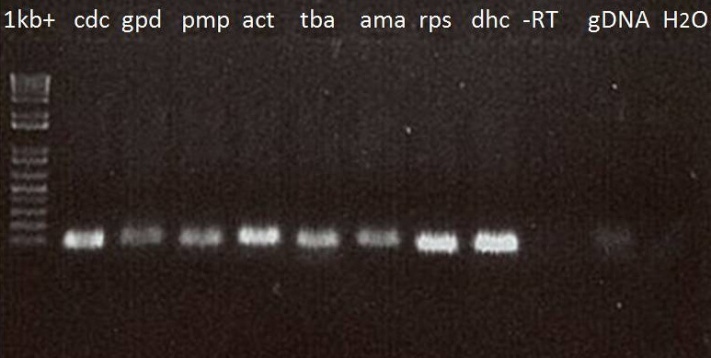


**Figure A:** Agarose gel showing single bands of the expected size for PCR products using 8 primer pairs, no band in the -RT control, a very faint band in the gDNA control and no band in the water only control.

Melting curves (step f in the qPCR program) were also performed for each individual gene. They were analysed for primer specificity (indicated by a single melting peak) and are listed below.

Efficiency assay: Standard curves were performed for each individual gene (technical triplicates) using a dilution series of cDNA (1×, 10×, 100×, 1000×, 10000×). For a qPCR assay the efficiency should be between 90% and 110%. This means that for every PCR cycle there is a doubling of the amount of product. Primers with efficiencies outside the acceptable range were discarded. qPCR primers and their efficiencies are listed in Table S1 and efficiency assays are listed below.

In each graph the amplification data is plotted with the log cDNA concentration on the x-axes and the threshold cycle on the y-axes. The threshold cycle is the cycle at which the fluorescence signal is first detected at higher than background levels. The software (BioRad FCX Manager) calculates efficiency (E), fit to the curve (R^2^) and slope of the curve (Slope). A R^2^ value between 0.95 and 1.0 indicates accurate efficiency measurement and a slope of -3.6 and -3.1 equals an efficiency of 90-100%.

*Pd-dhc*

*
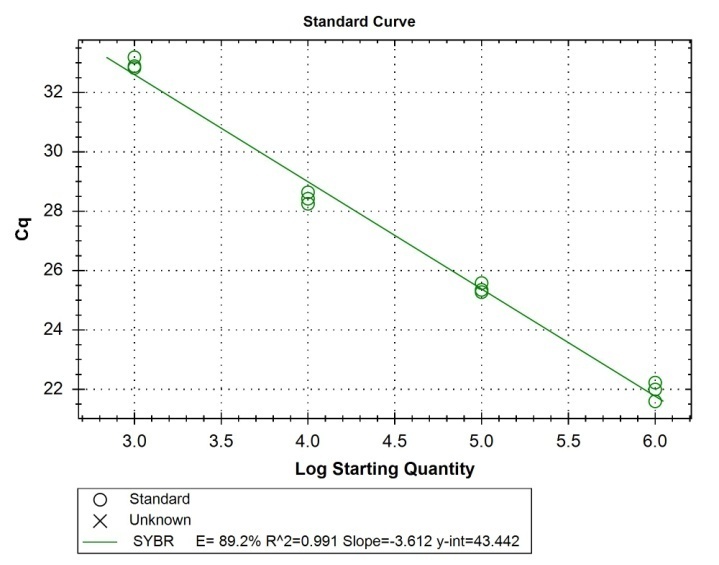

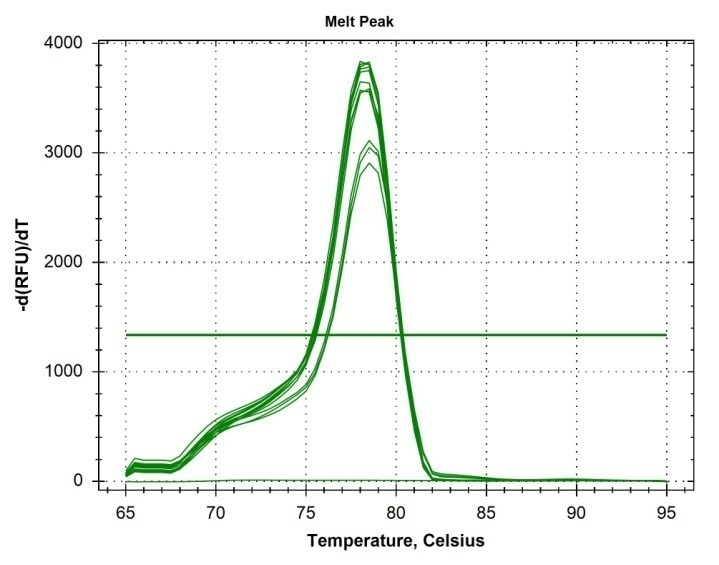
*

*Pd-duox-42*

*
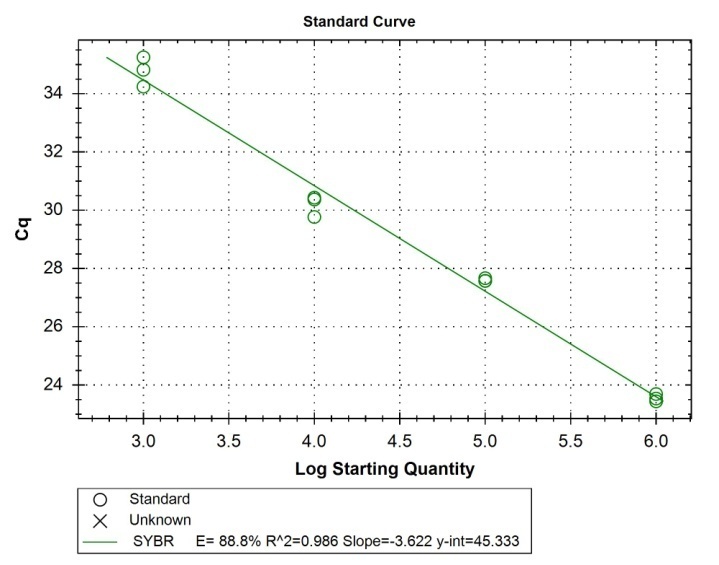

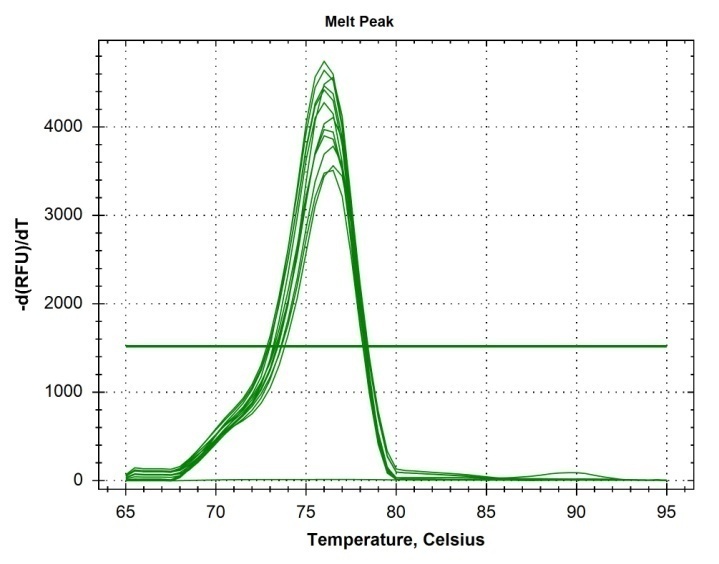
*

*Pd-gpd-2*

*
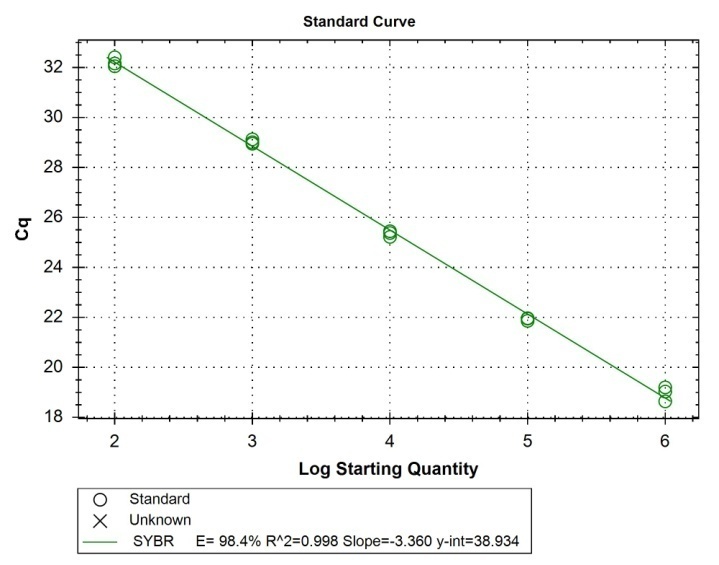

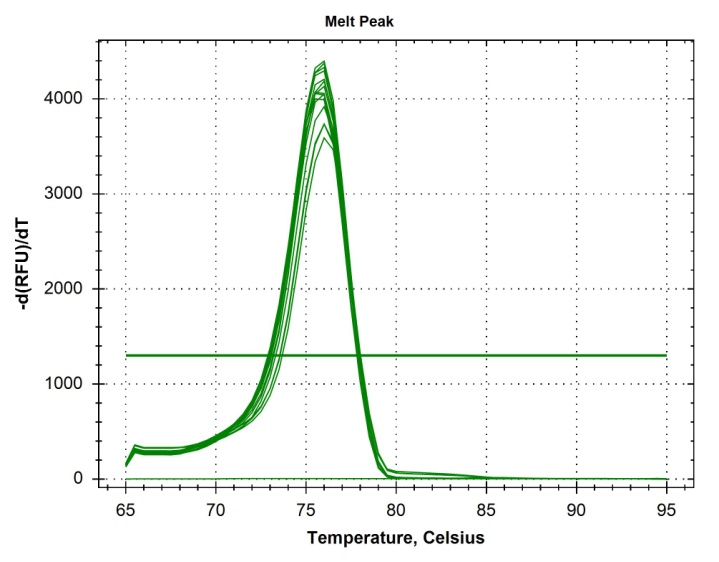
*

*Pd-rps-2*

*
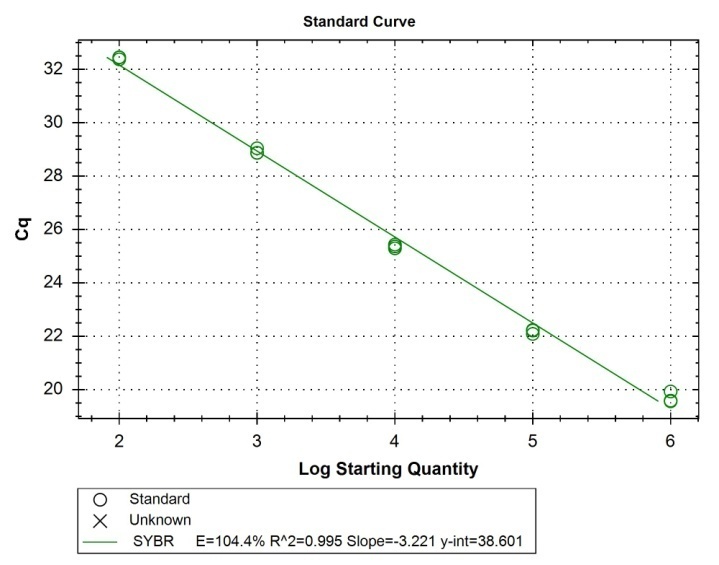

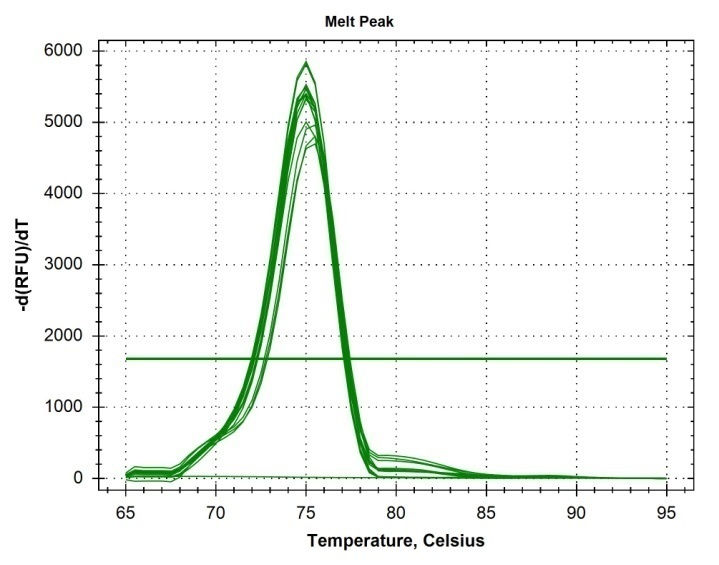
*

*Pd-tba-1*

*
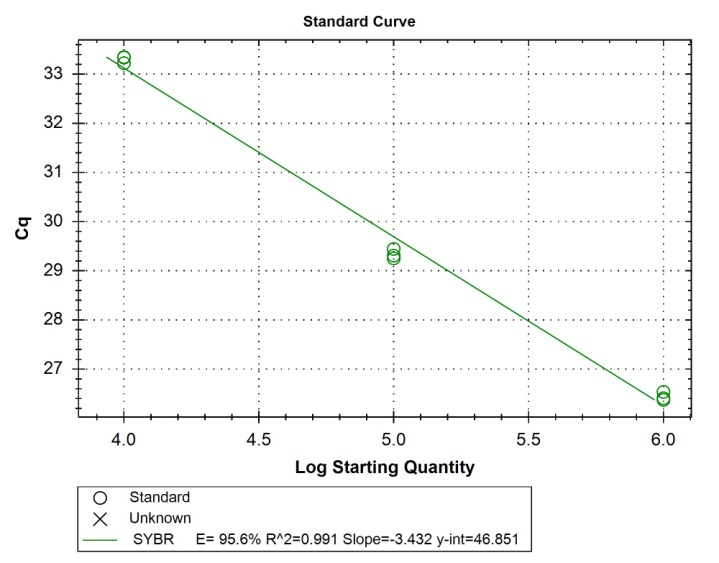

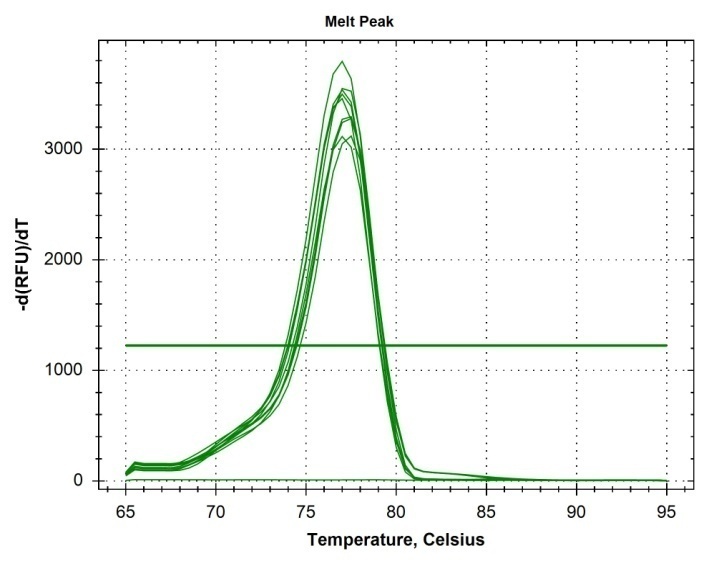
*

*Pd-tps-2a*

*
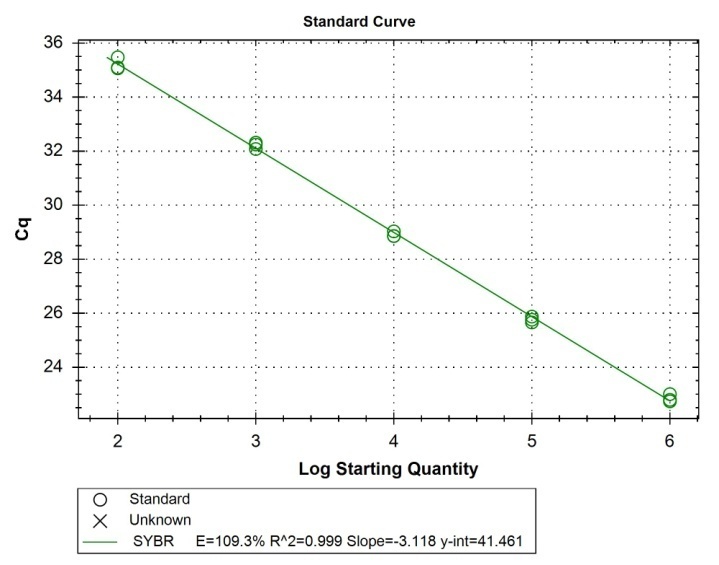

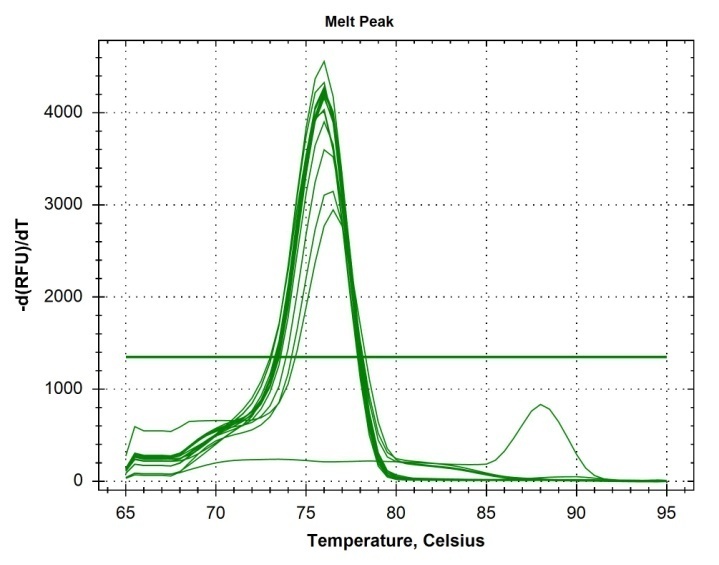
*

**Figure B.** Efficiency assays for each of the six genes used for qPCR analysis.

#### Reference genes

Reference genes for *P. davidi* were selected by searching the literature for stable reference genes in the closely-related *C. elegans*. Primers were designed for the following six reference genes (as described above): *Pd-act-1, Pd-ama-1, Pd-cdc-42, Pd-gpd-2, Pd-pmp-3* and *Pd-tba-1*. Of this group, the most stable combination of two reference genes were defined by using NormFinder (Table S2).

This algorithm estimates the stability of gene expression among groups, called the stability value. Genes that deviate the least from the calculated values are the most stable. With a stability value of 0.024, the combination of *Pd-gpd-2* and *Pd-tba-1* was defined as the most stable and used for all qPCR experiments. Because the suitability of selected reference genes can differ between experiments, the stability value in each experiment was analysed via NormFinder(Bustin et al., 2009).

| **Gene name** | **Stability value** |  | **Best gene** | |  |  |  | *Pd-gpd-2* |
| --- | --- | --- | --- | --- | --- | --- | --- | --- |
| *Pd-cdc-42* | 0.049 |  | **Stability value** | |  |  |  | 0.027 |
| *Pd-gpd-2* | 0.027 |  |  |  |  |  |  |  |
| *Pd-pmp-3* | 0.057 |  | **Best combination of two genes** | | | |  | *Pd-gpd-2* |
| *Pd-act-1* | 0.048 |  |  |  |  |  |  | *Pd-tba-1* |
| *Pd-tba-1* | 0.042 |  | **Stability value for best combination for two genes** | | | | | 0.024 |
| *Pd-ama-1* | 0.031 |  |  |  |  |  |  |  |

**Table C:** Stability value of six chosen reference genes for *P. davidi*: *Pd-cdc-42*, *Pd-gpd-2*, *Pd-pmp-3*, *Pd-act-1*, *Pd-tba-1* and *Pd-ama-1*. The most stable gene is *Pd-gpd-2* (stability value 0.027) and the most stable gene combination is *Pd-gpd-2* and *Pd-tba-1* (stability value 0.024).

These data are derived from 1. Seybold A. Molecular Adaptation Mechanisms in the Antarctic Nematode *Panagrolaimus davidi*. PhD Thesis, University of Otago, 2015.

**Reference**

1. Thorne MAS, Kagoshima H, Clark MS, Marshall CJ, Wharton DA. Molecular analysis of the cold tolerant Antarctic nematode, *Panagrolaimus davidi*. PLoS ONE. 2014;9: e104526–e104526. doi:10.1371/journal.pone.0104526
